# Patient Preferences for Receiving Remote Communication Support for Lifestyle Physical Activity Behaviour Change: The Perspective of Patients with Musculoskeletal Disorders from Three Hospital Services

**DOI:** 10.1155/2015/390352

**Published:** 2015-09-30

**Authors:** Steven M. McPhail, Mandy Schippers, Carol A. Maher, Alison L. Marshall

**Affiliations:** ^1^Centre for Functioning and Health Research, Metro South Health, Corner of Ipswich Road and Cornwall Street, Buranda, Brisbane, QLD 4102, Australia; ^2^Institute of Health and Biomedical Innovation and School of Public Health and Social Work, Queensland University of Technology, Victoria Park Road, Kelvin Grove, Brisbane, QLD 4059, Australia; ^3^Alliance for Research in Exercise, Nutrition and Activity, Sansom Institute, University of South Australia, North Terrace, Adelaide, SA 5000, Australia

## Abstract

This study examined patients' preference ratings for receiving support via remote communication to increase their lifestyle physical activity.* Methods*. People with musculoskeletal disorders (*n* = 221 of 296 eligible) accessing one of three clinics provided preference ratings for “how much” they wanted to receive physical activity support via five potential communication modalities. The five ratings were generated on a horizontal analogue rating scale (0 represented “not at all”; 10 represented “very much”).* Results*. Most (*n* = 155, 70%) desired referral to a physical activity promoting intervention. “Print and post” communications had the highest median preference rating (7/10), followed by email and telephone (both 5/10), text messaging (1/10), and private Internet-based social network messages (0/10). Desire to be referred was associated with higher preference for printed materials (coefficient = 2.739, *p* < 0.001), telephone calls (coefficient = 3.000, *p* < 0.001), and email (coefficient = 2.059, *p* = 0.02). Older age was associated with lower preference for email (coefficient = −0.100, *p* < 0.001), texting (coefficient = −0.096, *p* < 0.001), and social network messages (coefficient = −0.065, *p* < 0.001).* Conclusion*. Patients desiring support to be physically active indicated preferences for interventions with communication via print, email, or telephone calls.

## 1. Introduction

As an underlying cause of mortality and morbidity with an associated substantial economic burden, physical inactivity is a major international health concern [1–4]. People with musculoskeletal disorders have been specifically identified as a clinical population that may be at high risk of being insufficiently physically active, overweight, or obese and having multiple health conditions that may act as barriers to living a physically active lifestyle [5–8]. Physical inactivity, musculoskeletal disorders, obesity, and other chronic health conditions also increase in prevalence with chronological age, which is concerning given the increasing proportions of older adults in populations internationally [7, 9, 10].

The potential benefits of inactive people with musculoskeletal disorders becoming more physically active are broad ranging [11]. Higher lifestyle physical activity levels have been associated with lower risk of cardiovascular disease, respiratory benefits, improved mental health, and reduced mortality [3, 6, 11–14]. In addition to general health benefits, physical activity has also been associated with specific benefits to the musculoskeletal system which may include improved muscle strength and endurance, reduction in loss of bone mineral density, improved physical functioning among older adults, and lower risk of musculoskeletal pain in some conditions [11, 15–19]. However, individuals with musculoskeletal disorders may face additional barriers to becoming physically active compared to otherwise well members of society, including pain and fear of exacerbating their existing condition(s) [8, 20].

The point of interaction between health professionals and inactive patients with musculoskeletal disorders offers a unique opportunity to initiate lifestyle behaviour change interventions [7, 14]. However, for lifestyle behaviour change interventions to be successful among clinical populations, typically sustained interactions, engagement, and ongoing support are required [14, 21]. This is not always feasible during conventional consultations in busy clinical settings dependent on face-to-face interactions. As a result, clinician-patient interactions during time-limited consultations typically focus on mitigating the symptoms of the primary presenting complaint with cursory attention given to underlying contributory lifestyle factors [22–25]. While health professionals may like to assist patients to improve their health-related lifestyle profile, it has been suggested that this may not be feasible within the constraints of traditional face-to-face consultations [14, 26].

The recent advancements and rapid uptake of remote communication technologies offer new and flexible opportunities for supporting patients accessing health services to undertake physical activities appropriate for their existing conditions in the context of their daily lives [27]. The use of contemporary communication technologies for supporting physical activity behaviour change has demonstrated promise in a range of previous investigations [27–34]. Remote communication modalities that may have utility for use in interventions that promote physical activity targeted to people with musculoskeletal disorders may include email communication, short-message service (text messages to cell phones), and private messages on a web-based social network platform, in addition to more traditional voice telephone communication or printed materials sent via post. Remote communication modalities acting as a conduit for support mechanisms within physical activity behaviour change programs may offer flexibility in the timing of communication, the potential for automation, and preset communication templates, as well as the ability for messages to be received at an appropriate time in the context of the patient's own life [14, 31, 35, 36].

Although contemporary remote communication modalities may be a logical step in the evolution of physical activity interventions, patients' preference ratings for receiving remote communication for this purpose have yet to be examined. Patients with musculoskeletal disorders may or may not like to receive remote communication to assist them in living a physically active lifestyle. Similarly, they may have stronger preferences for particular communication modalities, and these preferences may be associated with personal attributes including their age, gender, whether or not they are already physically active, and whether or not they would like to participate in an intervention that aims to increase their physical activity levels. Understanding these patient preferences would be likely to benefit health professionals and researchers who seek to establish engaging and effective communication strategies for supporting patients with musculoskeletal disorders to safely increase their physical activity levels.

This study had two aims that were investigated among patients accessing hospital-based ambulatory clinics (nonsurgical) for musculoskeletal disorders. The first aim was to describe patients' preference ratings for five possible modalities for receiving remote communication support to increase their lifestyle physical activity behaviours. The five communication modalities to receive preference ratings from patients included print materials sent in the post, conventional telephone calls (voice calls), email communication, short-message service to their mobile phones (text messages to cell phones), or private messages on an online social network. The second aim was to examine whether several patient factors were related to patient preference ratings for each of these five modalities. Patient factors to be examined included age, gender, body mass index (BMI), whether the patients indicated that they wanted to be referred to a physical activity promoting intervention, and whether the patient self-reported physical activity levels that exceeded minimum recommended guidelines.

## 2. Methods

### 2.1. Design

A questionnaire battery including standardized measures and custom preference rating items was implemented among a cross section of patients accessing (one of) three hospital outpatient services.

### 2.2. Participants and Setting

Participants (*n* = 221) were consenting patients from a cross section of 296 community-dwelling adults accessing ambulatory hospital services for nonsurgical treatment of one or more musculoskeletal disorders. The ambulatory hospital services included a musculoskeletal physical therapy outpatient clinic, an orthopaedic screening clinic and multidisciplinary (physical therapy, psychology, occupational therapy, and nutrition and dietetics) service, and a musculoskeletal outpatient aquatic physical therapy service. These services were selected for this study as they were the three clinics for nonsurgical musculoskeletal interventions available to patients referred to the tertiary hospital facility in the metropolitan region where the study was conducted. These services were selected to represent a cross section of community-dwelling individuals with musculoskeletal conditions receiving nonsurgical interventions who may benefit from improved lifestyle physical activity [7]. All patients attending one or more of the clinics were eligible to participate. The only exclusion criterion was having already participated in the study (e.g., if a participant presented to a second participating clinic). It was intended that using a broad patient selection strategy in this way would increase the likelihood that results from this study could be extrapolated to this clinical population and similar health services. Patients were provided with a study information and consent form and a copy of the questionnaire which they could choose to return in hard copy to a participating clinic, enter their responses online via a web-based survey platform, or provide their responses to a research assistant via the telephone. Participation was voluntary. This investigation was approved by the Metro South Human Research Ethics Committee and the Queensland University of Technology Human Research Ethics Committee.

### 2.3. Outcomes and Procedure

The first components of the questionnaire battery included clinical and patient characteristics (primary reason for attending the clinic, age, and gender as well as height and weight which were used in the body mass index calculation). To determine whether patients self-reported physical activity levels that exceeded physical activity guidelines or not, patients were asked to report the frequency and duration of any walking or moderate-to-vigorous intensity physical activity they had completed in the past seven days using the Active Australia Survey [37]. Responses were combined to determine the individuals' total weekly activity in terms of frequency and duration [37]. For this calculation, vigorous physical activity time is assigned a “double” time weighting [37]. The accumulation of at least 5 sessions and 150 minutes (total) of moderate-to-vigorous physical activity was required to have met the minimum physical activity recommendations [37]. Patients were also asked whether or not they would like to be referred to a program to help increase their physical activity levels, if such a program was available to them (yes or no response).

Patients were subsequently provided with a statement explaining that several communication modalities were being examined for potential use in a program to assist patients to live physically active lifestyles. It was stated that the program would involve initial face-to-face contact with patients by a health professional running the program, with subsequent communication support offered via either (1) print materials sent in the post, (2) telephone calls, (3) email messages, (4) short-message service (also known as texting or text messaging), or (5) private messages sent via an online social network (e.g., Facebook). The statement also explained that any such program would be in addition to the existing health services and not a replacement or alternative for routine clinical care for patients. Patients were then asked to indicate their preference for each of the support modes by rating “how much” they would like to have a lifestyle physical activity support program made available to them on a horizontal analogue rating scale where 0 represented “not at all” and 10 represented “very much.” One preference rating was provided for each of the aforementioned communication support modes (five preference rating scores in total using the same rating scale).

### 2.4. Analysis

Analyses were conducted using STATA MP version 13. Patient clinical and demographic characteristics were described using conventional descriptive statistics, including number (and percentage), mean (and standard deviation), and median (and interquartile range). To address Aim 1, median and interquartile range were used to describe patients' preference ratings for each of the five potential modalities for receiving remote communication support as part of a lifestyle physical activity intervention. This was calculated for the whole sample and for each individual participating clinic. To address Aim 2, generalized linear mixed models were fitted. Patient preference ratings for the five communication modalities (print, telephone, email, SMS, and online social network private messages) were used as the dependent variable in each model. Fixed effects in each model included age, gender, body mass index, whether the patients wanted a lifestyle physical activity support program made available to them, and whether or not patients self-reported physical activity levels exceeded minimum recommended guidelines. An a priori interaction term between meeting minimum physical activity guidelines and wanting to be referred to a physical activity support intervention was also included in the model as a fixed effect. However, this interaction term was not significant in any model and was subsequently removed to reduce model complexity. It is noteworthy that the study was not designed or intended to specifically investigate variation in preference ratings between clinics; however, the multiple-level (two-level) nature of the data (patients nested in one of three services) was accounted for as a random effect (random slope) to honor the structure of the data. Due to the nature of the rating scale dependent variable and data structure, potential model parameters were examined to determine the most appropriate model using the Akaike information criterion as an indicator of model fit, which includes a penalty for complexity to avoid overfitting the model with complex parameterization. The Akaike information criterion consistently indicated that the generalized linear mixed model with Gaussian family and identity link function structure produced the best explanatory models across all five dependent variables with the aforementioned fixed and random effects (the final models are presented). However, the findings were consistent, albeit with a poorer model fit, when alternative family and link function structures were examined.

## 3. Results

The questionnaire battery was completed by all consenting participants (*n* = 221, 75% of eligible patients) with similar gender and age distributions across the three participating clinics (Table 1). The mean (standard deviation) age was 53 (15) years and 116 (52%) were male. The median (interquartile range) BMI for the sample was 28.3 (23.8–32.8), with 52 (23.5%) classified as overweight (BMI range 25–30) and 144 (41.6%) as obese (BMI > 30) [38]. The primary reasons for clinic attendance included musculoskeletal disorders affecting a spectrum of body regions, with the back (*n* = 84, 38%) and shoulder (*n* = 38, 17%) being the most frequently reported body regions affected. Approximately half (*n* = 112, 50%) of the participants reported insufficient physical activity relative to recommended guidelines.

Most (*n* = 155, 70%) of participants reported that they would like to be linked with a physical activity promoting intervention that included remote communication to assist them to be more physically active in their everyday lives. Participants' preference ratings for potential communication support modalities as part of the physical activity promoting intervention yielded a range of responses (Table 2). Printed communications sent via the post had the highest median preference rating (7 out of 10), followed by email and telephone (both with a median of 5 out of 10). Text messaging (median 1 out of 10) and private messages sent via an Internet social network (median 0 out of 10) had the lowest median preference ratings.

Factors associated with participants' preference ratings were not consistent across each of the potential communication modalities (Table 3). A desire to be referred to a physical activity promoting intervention was associated with higher preference for receiving remote communication via printed materials sent in the post (coefficient = 2.474, *p* < 0.001), telephone voice calls (coefficient = 1.854, *p* < 0.001), email (coefficient = 1.346, *p* = 0.02), and online social network private messages (coefficient = 0.824, *p* = 0.05). Older participant age was associated with lower preference for the three least traditional modes of communication, email (coefficient = −0.101, *p* < 0.001), texting (coefficient = −0.096, *p* < 0.001), and online social network private messages (coefficient = −0.065, *p* < 0.001). Higher BMI was associated with higher preference for online social network private messages (coefficient = 0.078, *p* < 0.01). Being insufficiently physically active (i.e., not meeting physical activity guidelines) was associated with higher preference for telephone voice calls (coefficient = 1.256, *p* = 0.01).

## 4. Discussion

This has been the first investigation of preferences for receiving remote-access communication support as part of a lifestyle intervention for patients accessing ambulatory hospital services for musculoskeletal disorders. The findings indicated that patients' preference ratings varied across the five potential communication modalities. The most traditional communication modality (print materials in the post) received the highest median preference rating followed by email and voice telephone calls which received the same median preference rating from this sample. The most novel potential communication modalities, SMS (texting) or private messages sent through an online social network, received low preference ratings. This suggests that patients from this clinical population may be unlikely to engage with either SMS or online social network private messages as a communication support mechanism as part of an intervention aiming at increasing lifestyle physical activity.

Patient age and desire to be referred to a lifestyle physical activity intervention were the two predominant patient characteristics associated with patient preference ratings, although these associations were not consistent across the five modality types. It was not surprising that a desire to participate in a physical activity program was associated with higher preference ratings for some modalities (receiving communication via print materials, telephone calls, emails, and private messages through an online social network). The association between older age and lower preference ratings for the communication technologies that have gained popularity more recently was also consistent with prior research investigating frequency of technology use among older adults [39]. It was noteworthy that no interaction existed between self-report of meeting minimum physical activity guidelines and desire to be referred to a physical activity intervention in any of the preference rating models. While the authors had no particular hypothesis regarding this potential interaction, such an interaction was considered plausible and worthy of investigation.

There are several implications from this study for lifestyle intervention development and planning among this clinical population. The preference ratings provided, and patient factors associated with these ratings, provide new insight when considering communication modalities that have potential for integration into interventions that promote physical activity. Interventions promoting physical activity with communication via print materials sent in the post, email, or telephone calls are worthy of further investigation, particularly among patients who are interested in being referred to a physical activity promoting intervention. In contrast, these findings suggest that caution is warranted when considering the potential use of novel communication technologies like SMS or online social networking as part of interventions that promote physical activity for middle-aged or older people with musculoskeletal disorders. While email was one of the most preferred communication modalities for the entire sample, it was less preferred among older adults who may be targeted for interventions promoting physical activity on account of their lower physical activity levels and presence of chronic health conditions that may benefit from physical activity [7].

Important limitations of this study were that it solely addressed the research aims related to investigating patient preferences, did not investigate broader socioeconomic factors, and was dependent on a description of intervention support mechanisms and patient reported information. This study does not provide information regarding efficacy or reasons for the reported preference ratings. Mobile phone ownership status and online social network usage were not collected. In addition, it is possible that the most preferred communication modalities may be perceived as familiar and convenient, which may not equate to efficacy. On the other hand, it is plausible that preferred communication modalities may be more likely to lead to the effective supportive communication required for behaviour change interventions to be successful. However, in the absence of an appropriately designed investigation of efficacy (or effectiveness), it is not yet possible to draw conclusions in this regard. Similarly, the least preferred communication mechanisms may or may not be perceived so unfavourably if patients were to experience them first hand as part of a physical activity support intervention.

There are also several factors that limit the ability to extrapolate findings from this study beyond similar populations. This investigation was undertaken specifically among a cross section of patients accessing outpatient hospital services for musculoskeletal disorders. Dissimilar patient groups may not have reported similar responses. Similarly, this investigation was undertaken in an industrialised nation where mobile telephone ownership and Internet access rates are very high and postal services are reliable [40]. Clinical groups from dissimilar societies may not have reported comparable findings.

A key priority for future research related to this topic is to evaluate the potential efficacy (or effectiveness) of interventions that promote physical activity among patients with musculoskeletal disorders using an appropriate clinical trial methodology. Findings from this investigation suggest that suitable remote communication modalities for potential integration into interventions that promote physical activity may include print materials in the post, email, and traditional telephone calls. However, the mode of communication may be considered the conduit for information to be conveyed and perhaps secondary to the supportive informational content that is being communicated. To this end, the development or refinement of existing content within physical activity behaviour change interventions [29, 30, 34] that could be implemented among patients with musculoskeletal disorders is also an important consideration. It would be beneficial for future research to also evaluate patients' lived experiences of participating in these interventions, in addition to the effect of interventions on clinical and physical activity outcomes.

## Figures and Tables

**Table 1:** Patient characteristics for the total sample and separately for each service.

	All participants (*n* = 221)	Musculoskeletal physical therapy clinic (*n* = 127)	Screening clinic and multidisciplinary service (*n* = 50)	Aquatic physical therapy (*n* = 44)
Age mean (SD)	53 (15)	51 (15)	53 (13)	59 (13)
Gender male *n* (%)	116 (52%)	65 (51%)	27 (54%)	24 (54%)
Body mass index median (interquartile range)	28.3 (23.8–32.8)	27.8 (23.7–31.7)	26.3 (23.9–33.8)	30.6 (24.1–34.1)
Sufficiently physically active *n* (%)	112 (50%)	71 (56%)	18 (36%)	23 (52%)
Desired referral to PA program *n* (%)	155 (70%)	79 (62%)	41 (82%)	35 (79%)
Primary presenting condition				
Back condition (%)	84 (38%)	34 (27%)	36 (72%)	14 (32%)
Shoulder condition (%)	38 (17%)	29 (23%)	4 (8%)	5 (11%)
Knee condition (%)	19 (9%)	11 (9%)	1 (2%)	7 (16%)
Neck condition (%)	18 (8%)	14 (11%)	4 (8%)	
Elbow, wrist, or hand condition (%)	12 (5%)	12 (9%)		
Ankle condition (%)	10 (5%)	7 (6%)	1 (2%)	2 (5%)
Hip condition (%)	8 (4%)	2 (2%)	2 (4%)	4 (9%)
Weight loss (%)	1 (<1%)	1 (1%)		
Other conditions (%)	31 (14%)	17 (13%)	2 (4%)	12 (27%)

**Table 2:** Median and interquartile range (IQR) preference rating for each of the five communication modalities (0 lowest, 10 highest).

	All participants (*n* = 221)	Musculoskeletal physical therapy clinic (*n* = 127)	Screening clinic and multidisciplinary service (*n* = 50)	Aquatic physical therapy (*n* = 44)
	Median (IQR)	Median (IQR)	Median (IQR)	Median (IQR)
Printed materials	7 (4–10)	6 (3–10)	7 (5–10)	8 (5.5–10)
Telephone	5 (0–7)	5 (0–7)	4.5 (0–7)	5 (0–8.5)
Email	5 (0–9)	5 (0–9)	4.5 (0–8)	3 (0–9)
SMS (texting)	1 (0–5)	1 (0–7)	2 (0–5)	0 (0–5)
Social network private messages	0 (0–2)	0 (0–2)	0 (0–2)	0 (0–0)

**Table 3:** Analyses including a summary of coefficients, their 95% confidence intervals, and *p* values from the generalized linear model for each communication modality (*n* = 221) with nonsignificant interaction terms omitted from the models.

Model dependent variable, Wald chi-square, *p* value	Independent variables (and interactions)	Coefficient	Coefficient 95% confidence intervals	*p* value
Print Wald *χ* ^2^(5) = 33.7, *p* < 0.001	Age	0.022	−0.009, 0.053	0.16
	Gender	0.698	−0.208, 1.610	0.13
	BMI	−0.005	−0.070, 0.061	0.89
	Desired support	2.474	1.482, 3.466	<0.001^*∗*^
	Physically active	−0.536	−1.476, 0.405	0.26

Telephone Wald *χ* ^2^(5) = 24.5, *p* < 0.001	Age	−0.016	−0.048, 0.016	0.32
	Gender	0.647	−0.272, 1.565	0.17
	BMI	0.013	−0.053, 0.080	0.70
	Desired support	1.854	0.833, 2.874	<0.001^*∗*^
	Physically active	−1.256	−2.222, −0.289	0.01^*∗*^

Email Wald *χ* ^2^(5) = 37.7, *p* < 0.001	Age	−0.101	−0.137, −0.066	<0.001^*∗*^
	Gender	0.076	−0.956, 1.107	0.87
	BMI	0.026	−0.049, 0.100	0.50
	Desired support	1.346	0.201, 2.249	0.02^*∗*^
	Physically active	0.358	−0.727, 1.444	0.52

SMS (texting) Wald *χ* ^2^(5) = 41.1, *p* < 0.001	Age	−0.096	−0.126, −0.065	<0.001^*∗*^
	Gender	0.306	−0.590, 1.202	0.50
	BMI	0.010	−0.055, 0.075	0.75
	Desired support	0.780	−0.201, 1.761	0.12
	Physically active	−0.537	−1.467, 0.394	0.26

Social network private messages Wald *χ* ^2^(5) = 34.0, *p* < 0.001	Age	−0.065	−0.090, −0.039	<0.001^*∗*^
	Gender	−0.138	−0.871, 0.593	0.71
	BMI	0.078	0.250, 0.131	<0.01^*∗*^
	Desired support	0.824	0.012, 1.636	0.05^*∗*^
	Physically active	0.090	−0.680, 0.860	0.82

Notes: the two-level nature of the data (patients nested in one of three clinics) was included as a random effect (random slope) in each model (not significant in any model). *∗* indicates being significant at alpha = 0.05.
